# President’s Address, Illinois State Veterinary Medical Association1Presented at the annual meeting by Dr. I. J. Gunning, President, November, 1901.

**Published:** 1902-01

**Authors:** 


### PRESIDENT'S ADDRESS, ILLINOIS STATE VETERINARY MEDICAL ASSOCIATION.[1]

ANOTHER year has passed since last we met together at the annual meeting of this Association. The past, like all other years, has brought joy to some and sorrow to others. It is only a few weeks since our great nation was called to mourn the loss of its chief Executive—a man greatly beloved by all except by the Anarchists. Surrounded by his fellow-citizens, vieing with each other to show him honor, he was laid low by the assassin's bullet, and the name of William McKinley was added to the list of martyred Presidents; and while we bow in humble submission to the will of the Almighty, and mourn the loss of one so grand and noble, let us not forget to render thanks to Him who doeth all things well.

We have just reasons to be thankful when we remember that during the past year none of our members has been called from time to eternity, and our ranks remain unbroken.

I wish to acknowledge my indebtedness to our worthy Secretary for the able support he has given me during the past year. I can

[1] Presented at the annual meeting by Dr. I. J. Gunning, President, November, 1901.

assure you that had it not been for his untiring efforts—his planning, pleading, and begging—the programme of this meeting would not present such an array of well-known names, which is a guarantee that we will receive papers of interest to all.

The office of Secretary should be filled by the best man in the Association, and I can assure you that there was no mistake made when Dr. Welch was chosen our Secretary; but, like some political parties, the mistake was made at the head of the ticket, and I trust that you will profit by the present experience, and do better in the future in selecting a man to act as our President.

During the past year there has been no serious outbreak of contagious or infectious diseases in the State, so far as I have been informed, unless it be influenza, which has been quite prevalent, but of a mild form, the death-rate being quite small, most of the deaths occurring in neglected cases. Where there has been an outbreak of disease it has been confined to a small section of the State. Some of these local outbreaks have not been reported, many of the stockmen of the State having lost all confidence in the work done by the State the past four years, and will not consent to State interference if they can prevent it.

The past year has been one of prosperity for the veterinarian as well as the stockmen of the State. The advanced price of livestock has made owners of stock more interested in caring for their sick animals, and, money being quite plentiful, the doctor has received more cash for his work and fewer accounts to charge and collect in the future.

The prosperity enjoyed by the profession during the past two years will undoubtedly cause many of the young men to start in the work of educating themselves for the profession, notwithstanding the fact that the Governors of this State persist in refusing to recognize the graduate veterinarian. Our colleges will receive more or less benefit from the present prosperity by a larger number of students, and the colleges offering the most complete course of study and receiving the most public recognition will without doubt receive the largest number of students. Our colleges should be the standard-bearers of the profession, and should at all times refuse to recognize any man or class of men who, by word or deed, strive to cast any reflection on the colleges of their graduates. The work of our colleges and the persistent work of scientific men in the past few years have elevated the scientific knowledge of the profession to a place worthy of the highest recognition of every American citizen.

As President of this Association I feel that there is a duty resting on me—a duty that I would gladly shun if by so doing I would feel that I was doing my duty to our profession. The duty that I refer to is the standing of our profession in this State, and I can assure you that if I did not feel it my duty to make a few remarks along this line I would gladly pass it by; and I can assure you that what I have to say I say with true brotherly love and due respect for all.

I trust that the time is not far distant when every graduate in this State will be bound together in our great professional brotherhood, thinking not of self alone, but the good of all. The profession in this State at the present time reminds me very much of a house divided against itself. What the outcome of this division will be it is not for me to say, but I trust that the divided parts may again be bound together for the good of all.

Something over four years ago the Governor of this State appointed to the office of State veterinarian a gentleman who was a non-graduate—a gentleman who, only a short time before his appointment, took occasion in my presence to denounce the veterinary colleges of this country as unworthy of recognition; and I took it for granted that he did not hold graduates in any higher esteem than the colleges from which they were graduated. Only a short time after this appointment he was in Chicago, knocking at the doors of the veterinary colleges, asking them to recognize him; and I have been informed that there was one of the veterinary colleges which gave him the recognition he desired, and which, I have no doubt, he so much needed, while the other college was perfectly willing to recognize him as an honorable gentleman, but not as a professional man, and by so doing placed a wreath around it which neither the scorching sun of summer nor the chilling blasts of winter will be able to wither.

At the time of this appointment it was freely admitted by State officials at Springfield that if the graduated veterinarians of this State refused to do State work under this appointment the State work could not be carried on; but if enough graduates could be found who would condescend to do State work it would make but little difference who held the office of State veterinarian. Notwithstanding the fact that a large number of graduates refused to do State work, there were plenty who seemed not only willing, but anxious, to have an appointment to do State work. The veterinary profession was in no way to blame for the first appointment of a non-graduate, the blame resting with a few of the political wire-pullers of the Republican party; but for the second appointment the graduates who made it possible for a non-graduate to hold the office are alone responsible, for by their work they have made it possible for any man to hold the office that a few wire-pullers may see fit to name.

During the past winter quite a number of graduates came out as candidates for the office of State veterinarian—men who would have been an honor to the office and to the profession—and, after obtaining indorsements of which they might feel justly proud, were each and all passed by without the least recognition. Some of these men had been serving the State for nearly four years, and after four years of work succeeded in doing just what they had been working for—their own defeat; and I can assure you I regret their defeat perhaps more than they do. It seems to me that if those men had been just a little better posted on politics there would have been very few candidates in the field, for it was a foregone conclusion at the time the present Governor received the nomination that if elected no graduate would be appointed.

There was never any doubt as to where the Democratic party or the candidates stood in regard to the appointment of a State veterinarian; but, strange as it may seem, out of the four candidates of the Republican party there was only one who dared to say one word as to where he stood on the appointment of State veterinarian; and I take pleasure in saying that the one Republican candidate who spoke with no uncertain sound for our schools and for the veterinary graduates of medicine and surgery was Hon. Judge Carter, of Chicago; and I wish to say to you that if this meeting adjourns without giving a vote of thanks to the defeated Democratic nominee and to Judge Carter it will adjourn without showing due respect for the men who were ready and willing to give just recognition to the graduates of this State.

It would be gross injustice to the present State veterinarian to say, as some have said, that he had no right to accept this office. He had the same right and privilege as any other citizen of this State; and just as long as he can find men who are willing to kneel at his footstool he is likely to hold his present office, and I shall be the last to criticise him for so doing.

Let me ask you how long you think the State work could be carried on if every graduate who is now doing State work would refuse to work, and all other graduates do likewise. Let me say to you that it would not be forty-eight hours until there would be a few men at Springfield hunting for the point of the compass to find out "where they were at;" and during that forty-eight hours the profession would rise to a place of honor and recognition such as it never has known in the State.

In the course of my remarks I may have said some things that would ruffle the feathers of some brother practitioner, but I believe that friendly criticism will often do more good than ordinary silence. I wish to say to you to-day that just as long as the graduates of this State are willing to submit to the dictates of political wire-pullers our colleges and our profession will never receive just recognition.

The live-stock interests of this State are ready and willing to do us justice and lend friendly assistance at any time, but we must first show that we stand united, and are making a strong pull and a pull altogether for the good of each and all.

I hope that the day is not far distant when every graduate in this State will stand under one banner and take as their watchword:

"We live for those who love us,
For those who know us true;
For the wrongs that need resistance,
For the rights that need assistance,
For the future in the distance,
And good that we can do."

